# New insights into swine dysentery: faecal shedding, macro and microscopic lesions and biomarkers in early and acute stages of *Brachyspira hyodysenteriae* infection

**DOI:** 10.1186/s40813-024-00375-9

**Published:** 2024-06-29

**Authors:** Lucía Pérez-Pérez, Ana Carvajal, Héctor Puente, Camila Peres Rubio, Jose Joaquín Cerón, Pedro Rubio, Héctor Argüello

**Affiliations:** 1 Departamento de Sanidad Animal, Facultad de Veterinaria, Universidad de León, León, Spain; 2 INDEGSAL, Universidad de León, León, Spain; 3 Interdisciplinary Laboratory of Clinical Analysis (Interlab-UMU), Campus de Excelencia Internacional Mare Nostrum, Universidad de Murcia, Murcia, Spain

**Keywords:** Swine dysentery, Pig, Acute phase proteins, Mucohaemorrhagic diarrhoea, Spirochaetes

## Abstract

**Background:**

Swine dysentery (SD) is a severe mucohaemorrhagic colitis in pigs caused classically by *Brachyspira hyodysenteriae*. Although several aspects of *B. hyodysenteriae* infection dynamic are already described, further research in the early stage of this infection is required. In this study, 7-week-old pigs were orally challenged with *B. hyodysenteriae* to obtain information about faecal shedding, macro and microscopic intestinal lesions and serum acute phase proteins in pigs at the onset of *B. hyodysenteriae* shedding (early infection group, *n* = 8), in pigs with mucohaemorrhagic diarrhoea (acute infection group, *n* = 8) and in non-infected controls (*n* = 16).

**Results:**

First *B. hyodysenteriae* detection by q-PCR and first loose stools with blood and mucus occurred both at 8 days post-inoculation. The lapse between a positive q-PCR and observation of mucohaemorrhagic diarrhoea ranged from 0 to 3 days, except in a single pig in which this period lasted 5 days. Macroscopic lesions were observed in the large intestine from both infected groups although more frequent and severe in acute infection group. Microscopic observation of the apex mucosa revealed that in early infection only higher ulceration values were observed compared to healthy controls. In contrast, the acute infection group exhibited higher ulceration, neutrophils infiltration and increased mucosal thickness compared to the other two groups. Among the serum biomarkers tested, only haptoglobin, C-reactive protein, and creatine kinase showed a significant increase in pigs in the acute infection period compared to controls, whereas haptoglobin was the only factor with a significant increase at the early infection compared to non-infected animals.

**Conclusions:**

This study provides new insights about SD and remarks the complex and limited options to perform an early detection of infected animals beyond PCR diagnosis.

**Supplementary Information:**

The online version contains supplementary material available at 10.1186/s40813-024-00375-9.

## Background

Swine dysentery (SD) is an enteric disease characterized by severe mucohaemorrhagic diarrhoea, mostly observed in grower-finisher pigs, and often resulting in significant economic losses associated to mortality, antimicrobial treatments and poor feed conversion [1]. Strongly beta-haemolytic spirochetes are the causative agents of this disease. *Brachyspira hyodysenteriae* is the classical etiological species but recently other two species have been associated to SD, *Brachyspira suanatina* and *Brachyspira hampsonii* [2, 3].

The importance of SD has encouraged research to shed light on its aetiology, epidemiology or control [4–6]. Despite the efforts over decades to gain knowledge about the disease, the statement of ter Huurne and Gaastra [7] “Swine dysentery: more unknown than known” is still in force. In the last two decades, several challenge studies under controlled conditions have been used to gain knowledge about SD [8–13]. There is a large variability in the approach used to design these studies, including the *Brachyspira* species and strains used, the challenging model (oral vs. intragastric gavage or shedder model), the pathogen detection method of choice or parameters measured in the monitoring of the disease outcome. Rather than limiting potential comparisons, this heterogenicity offers the opportunity to gather knowledge from different approaches and identify gaps which can be filled in subsequent studies. Early detection and diagnosis of SD is of paramount relevance to limit the infection transmission and mitigate its severity in infected batches, particularly, the detection of carriers or infected pigs that do not have clinical signs but shed the bacteria in faeces (early stage). Understanding the risk of shedding before clinical disease and potential tools to detect the early stages of infection are of particular interest. In this sense, serum biomarkers may be a tool useful in SD diagnosis, as previously pointed by two studies [12, 14]. Markers of inflammation, mostly acute phase proteins (APPs) and markers of oxidative stress such as ferric reducing ability of plasma (FRAP) or Thiol have been linked to several diseases and clinical conditions in pigs [15, 16]. Similarly, serum levels of triglycerides (TRIGL) or intracellular enzymes, such as creatine kinase (CK) have been used to assess syndromes that cause diarrhoea [17] or intestinal damage [18] respectively, closely related to SD.

The present study, using a non-hyperproteic diet driven *B. hyodysenteriae* challenge, evaluates the infection and disease outcome in pigs by the analysis of pathogen faecal shedding, macro and microscopic lesions in the large intestine and serum biomarker response in early and acute infection stages.

## Methods

### Experimental challenge with ***B. hyodysenteriae*** and clinical assessment of the infection

All procedures were approved by the University of León Committee on Animal Care and Supply (OEBA-ULE-010-2020).

Thirty-two 7-week-old crossbred (Landrace x LargeWhite x Pietrain) female pigs from a commercial farm with no history of SD or porcine spirochaetosis, were randomly assigned into two groups, challenged (*n* = 16) and control (*n* = 16). The groups were allocated into two isolated loose boxes within the biocontention facilities at the University of León with controlled light and temperature conditions and concrete floor. Pigs had free-access to water and were fed *ad-libitum* with a commercial non-medicated pelleted diet for growers. At arrival, pigs were individually ear-tagged and within 10-day acclimation period, each animal was confirmed negative for *B. hyodysenteriae* and *B. pilosicoli* by culture and PCR detection of the *B. hyodysenteriae* haemolysin regulatory gene tlyA and the *B. pilosicoli* 16S rRNA gene [19].

After the acclimation period, once daily, on three consecutive days (0, 1, and 2 days post-inoculation (DPI)), pigs from the challenged group were orally administered, using a feeding syringe, 30 mL of B-204 *B. hyodysenteriae* (ATCC 31212) pure culture in brain heart infusion broth (BHI) supplemented with 10% foetal bovine serum (FBS) containing 5 × 10^8^ spirochaetes per mL. Pigs in the control group received an equivalent volume of sterile BHI-10% FBS broth.

Faecal samples were collected daily from all pigs from day 0 (first sample collected just before *B. hyodysenteriae* challenge) onwards, by digital rectal stimulation. Straight after faecal sampling, the faecal consistency was recorded with the following score: 0 if normal, 1 if soft but formed, 2 if semisolid, and 3 if liquid to watery, with addition of 0.5 for the presence of detectable mucus and/or blood according to Wilberts et al. [8]. A fresh faecal subsample was kept at 4° C for *B. hyodysenteriae* culture and another subsample was frozen at -80° C for DNA extraction.

Half of the pigs in the challenged group (*n* = 8) were euthanized 24 h after the first positive q-PCR faecal sample (named early infection group). The rest of the challenged pigs (*n* = 8) were euthanized during the acute stage of infection, with q-PCR positive faeces and two consecutive sampling days with faecal score 3.5 (named acute infection group). Control pigs were euthanized and necropsied in parallel, one to one, with challenged pigs.

Immediately after euthanasia, serum samples were obtained by jugular vein puncture into vacuum tubes without additives. Upon necropsy, the entire intestinal tract was examined for macroscopic lesions, and the caecum and colon were evaluated for the presence of oedema, excessive luminal mucus, mucosal haemorrhage and fibrinous exudate. Colonic digesta was collected from an incision in the apex of the spiral colon and snap-frozen with liquid nitrogen. Thereafter, the apex of the spiral colon was removed, rinsed thrice with sterile phosphate-buffered saline (PBS) and fixed in 10% neutral buffered formalin.

### Detection and quantification of ***B. hyodysenteriae***

Faecal detection of *B. hyodysenteriae* by culture was performed as described elsewhere [20]. Briefly, faecal samples were cultured on trypticase soy agar (TSA, Scharlab, Spain) supplemented with 5% sheep blood (Oxoid, Spain), 400 μg/mL spectinomycin, 8 μg/mL colistin and 20 μg/mL vancomycin (Sigma-Aldrich, United States) at 40° C under anaerobic conditions. Positive cultures, after up to 7 days of incubation, were identified by zones of strong β-haemolysis from which motile spirochetes could be seen by phase-contrast microscopy. To specifically detect *B. hyodysenteriae*, DNA was obtained from the haemolytic growth in agar by freezing it in 50 μL of ultrapure water and used as template for PCR detection of the putative *B. hyodysenteriae* haemolysin regulatory gene tlyA [19].

In parallel, DNA from each faecal sample was extracted using a commercial kit (QIAamp^®^ PowerFecal^®^ Pro DNA Kit, Qiagen, Germany), according to the manufacturer’s instructions. Quantification *of B. hyodysenteriae* in the extracted DNA was carried out in a QuantStudio 1 thermal cycler (Applied Biosystems, United States), using *Brachyspira*-specific primers in combination with a *B. hyodysenteriae*-specific probe as previously described [21] and using an in-house standard curve to extrapolate cycle threshold (Ct) values to bacteria/g of faeces. The standard curve was prepared using ten-fold serial dilutions of a *B. hyodysenteriae* B204 pure culture (initial load 10^8^ bacteria/mL and range 10^8^-10^2^ bacteria/mL) and the detection limit was defined by the linear portion of the standard curve and was set at 10^3^ bacteria/mL (limit Ct detection at 35.7).

### Histopathology

After 48 h of fixation, the apex of the spiral colon was processed and stained with haematoxylin and eosin by standard procedures. Sections of each tissue were blindly evaluated by a veterinary pathologist for the presence of ulceration, haemorrhage, neutrophilic infiltration of the lamina propria and mucosal thickness according to Wilberts et al. [8]. Ulceration was scored as follows: 0 if no ulceration, 1 for focal ulceration spanning 1 to 3 crypts, 2 for focal ulceration spanning 3 to 5 crypts and 3 for focal ulceration spanning more than 5 crypts. An additional 0.5 was added for multifocal ulceration or 1 for multifocal ulceration spanning more than 5 crypts. Haemorrhage was evaluated upon the most severe lesion in the lamina propria and lumen, and assigned a score as follows: 0 if ≤ 5 red blood cells (RBC), 1 if 6 to 10 RBC, 2 if 11 to 20 RBC, 4 if 21 to 50 RBC, and 5 if ≥ 51 RBC with an additional 1 point added to the score for more than 3 foci of haemorrhage. The quantity of neutrophils in the lamina propria of the apex was determined estimating the mean of ten 40x fields. Finally, the mucosal thickness was calculated as the mean of three measurements from the area where the crypts were perpendicular to the mucosal surface with intact epithelium using a standard eyepiece micrometre.

### Blood biomarkers quantification

Total protein (PROT), albumin (ALBU), TRIGL and CK were measured by spectrophotometric assays using commercially available reagents (Beckman Coulter^®^, United States). Paraoxonase 1 (PON 1) was measured by an automated assay using 4-(p)-nitrophenyl acetate as substrate that has been previously validated in pig serum [22]. Adenosine deaminase (ADA), butyrylcholinesterase (BchE) and acetylcholinesterase (AchE) were measured by spectrophotometric assays also validated previously in pig serum [23, 24]. Thiol and FRAP were measured as described in Rubio et al. [25]. Haptoglobin (HAPTO) and C-reactive protein (CRP) levels were measured in serum by immunoturbidimetric assays with commercial reagents from SPINREACT, S.A.U (Spain) and Beckman Coulter^®^ (United States), respectively, tests already validated in pig serum samples [26]. All the assays were measured using an automated biochemistry analyzer (Olympus AU600 Automatic Chemistry Analyzer, Olympus Europe GmbH, Germany).

### Statistical analysis

Statistical analysis was performed in R v4.3.3 and plots were built using ggplot2 v3.5.0 [27, 28]. Faecal concentration of *B. hyodysenteriae* and quantitative data recorded from histopathology and serum parameters were assessed for normality by Shapiro Wilk’s test (R package stats v4.3.3 [27]) and based on data distribution statistical differences among groups were evaluated by one-way analysis of variance (ANOVA) or Kruskal-Wallis with a Tukey or Wilcoxon tests for multiple comparisons and Holm as an adjustment method (stats v4.3.3 [27] and ggpubr v0.6.0 [29] R packages). In all circumstances, values of *P* ≤ 0.05 were considered significant.

Correlation among *B. hyodysenteriae* faecal concentration with faecal score and histopathological lesions, as well as between histological lesions and serum protein levels was determined by Spearman’s rank order correlation or Pearson correlation coefficient according to the data distribution (R package ggpubr v0.6.0 [29]).

## Results

### Disease outcome and ***B. hyodysenteriae*** shedding in faeces

**Fig. 1:**
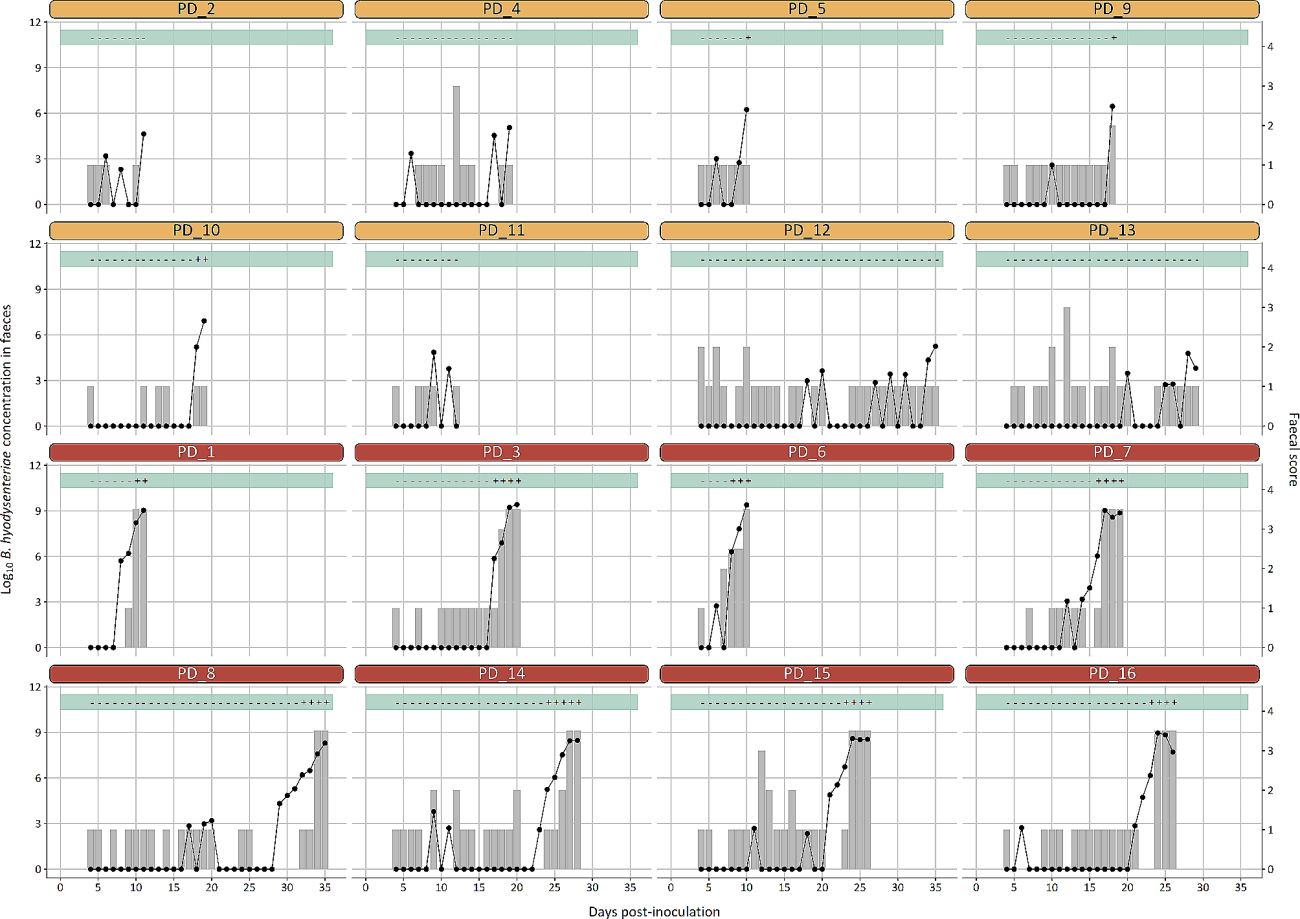
Summary of faecal monitoring of *Brachyspira hyodysenteriae* in challenged pigs. Dotted line shows the concentration of *B. hyodysenteriae* in faeces estimated by qPCR (left axes); grey graph bar shows the faecal score (right axes) according to Wilberts et al. [8] in each monitored day. Positive (+) and negative (-) results in the green bar over each graph summarise the results of microbiological culture of *B. hyodysenteriae*. Pig IDs are indicated at the top of each panel (early infection in yellow and acute infection in dark red)

The study lapsed 36 days from challenge (0 DPI) until the last pig shed the pathogen in faeces. According to q-PCR analyses, all challenged pigs excreted *B. hyodysenteriae* in their faeces, and excretion started on average 18.06 ± 7.93 DPI (Table 1; Fig. 1). The first detection of *B. hyodysenteriae* in faeces occurred at 8 DPI.

**Table 1:** Data of the animals, dates and q-PCR results from pigs challenged with *Brachyspira hyodysenteriae*

Group	Challenged pig	First q-PCR positive (DPI)	Concentration in last q-PCR positive result (bacteria/g of faeces)	First mucohaemorrhagic faeces (DPI)	Score in the last faecal sample	Euthanasia day (DPI)	Control pig
Early infection	PD2	11	4.37 × 10^4^	-	0	11	PC4
	PD4	17	1.13 × 10^5^	-	1	19	PC16
	PD5	10	1.76 × 10^6^	-	1	11	PC5
	PD9	18	2.89 × 10^6^	-	2	18	PC11
	PD10	18	8.35 × 10^6^	-	1	19	PC7
	PD11	9	5.95 × 10^3^	-	1	12	PC15
	PD12	34	1.78 × 10^5^	-	1	36	PC13
	PD13	28	6.35 × 10^3^	-	1	29	PC1
Acute infection	PD1	8	1.10 × 10^9^	10	3.5	11	PC3
	PD3	17	2.64 × 10^9^	19	3.5	20	PC8
	PD6	8	2.51 × 10^9^	8	3.5	10	PC6
	PD7	15	7.40 × 10^8^	17	3.5	19	PC14
	PD8	29	1.98 × 10^8^	34	3.5	36	PC2
	PD14	24	2.96 × 10^8^	27	3.5	28	PC12
	PD15	21	3.54 × 10^8^	24	3.5	26	PC10
	PD16	22	5.10 × 10^7^	24	3.5	26	PC9

DPI: Days post-inoculation

Pigs euthanized at early infection shed a mean concentration of *B. hyodysenteriae* of 1.67 ± 2.90 × 10^6^ bacteria/g of faeces with normal or semisolid faeces, while pigs euthanized in the acute stage of the disease shed 9.85 ± 10.35 × 10^8^ bacteria/g of faeces and diarrhoea with mucus and/or blood (*P* < 0.001) (Table 1; Fig. 1). The concentration of *B. hyodysenteriae* in faeces, estimated by q-PCR, correlated positively with faecal score (*R* = 0.87, *P* < 0.001). Microbiological cultures only got positive when the quantity detected by q-PCR exceeded 10^5^ bacteria/g of faeces (Fig. 1).

Faecal scores recorded the day of the first q-PCR positive in each animal varied from 0 to 1, except in a pig with semisolid faeces (score 2, #PD9) and another pig with mucus and blood (score 2.5, #PD6). The lapse between a positive q-PCR and the development of mucohaemorragic diarrhoea ranged from 0 to 3 days in all except one pig with 5 days (#PD8) (Fig. 1). Loose stools with blood and mucus were first observed at 8 DPI and it was at 34 DPI when the last pig got clinically sick, with a mean time to observe mucohaemorrhagic diarrhoea of 20.25 ± 8.91 DPI in pigs from the acute infection group.

None of the animals in the control group had diarrhoea (faecal score ≥ 2) during the study period and pigs in early infection group were euthanized before they could develop severe clinical signs, with faecal scores ranging between 0 and 2 (Table 1).

### Macro and microscopic lesions

Macroscopic pathological findings were consistent with clinical signs observed, except in one pig (#PD5) that presented a severely affected apex but faeces with normal consistency. Lesions were more frequent and seemed more severe in pigs within the acute infection than in early infection. The latest exhibited catarrhal typhlocolitis, while in the acute infection group hyperplasia of the mesenteric lymph nodes, meso-colonic oedema and/or congestion, mucohaemorrhagic and necrotic-fibrinous typhlocolitis and variable degrees of mucosal thickening were observed, particularly at the apex, which was the most severe affected colonic region.

**Fig. 2:**
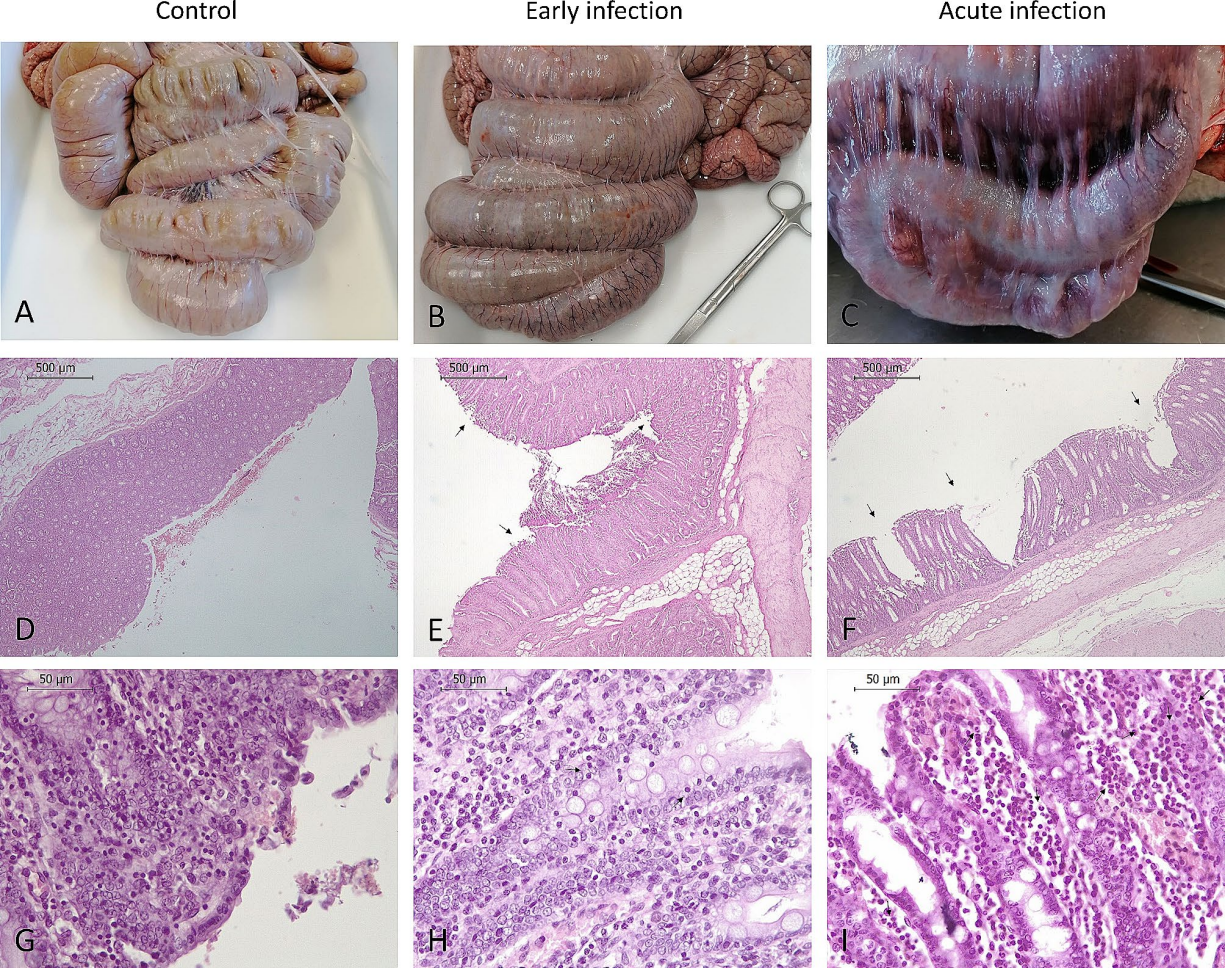
Colon and apex tissue sections from control and *B. hyodysenteriae* infected pigs. Macroscopic pathological findings: (**A**) Colon without macroscopic lesion (control); (**B**) Colon with mild hyperaemia (early infection); (**C**) Colon with severe hyperaemia and/or congestion, severe meso-colonic oedema and moderate hyperplasia of the mesenteric lymph nodes (acute infection). Photomicrographs at high and low magnifications of the apex stained with haematoxylin and eosin; (**D**) Normal mucosal apex (control); (**E**) Mild necrosis of the epithelium (early infection); (**F**) Severe necrosis of the epithelium (acute infection); (**G**) No lesion, only mild haemorrhage (control); (**H**) Moderate number of lymphocytes in the epithelium (early infection); (**I**) High number of neutrophils in the lamina propria (acute infection). Arrows point areas of ulceration and necrosis (2E and 2 F) and the inflamatory infiltrate (2 H and 2I)

**Fig. 3:**
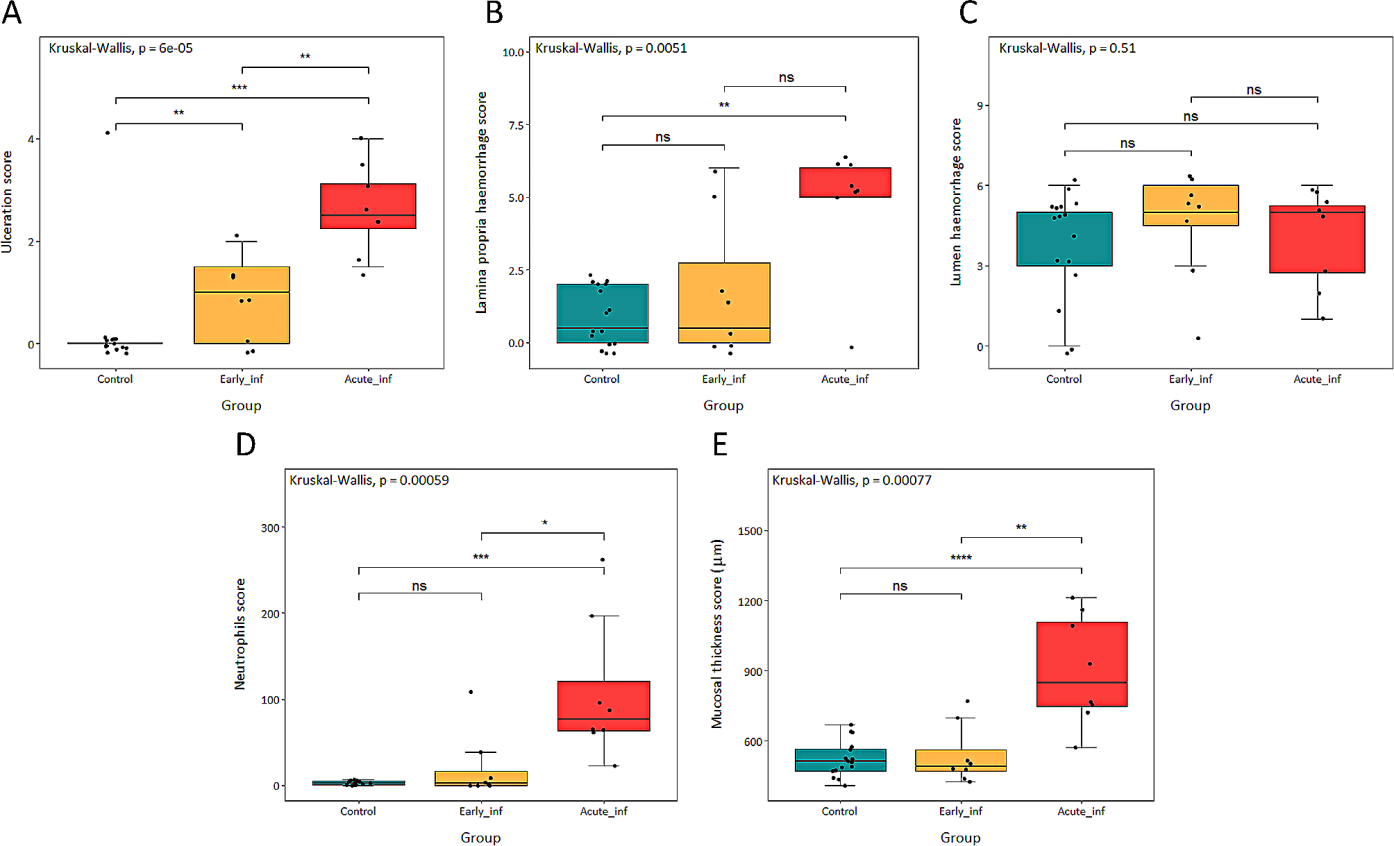
Boxplots of apex microscopic lesions in controls and *B. hyodysenteriae* infected pigs. (**A**) Ulceration scored as 0 = no ulceration; 1 = focal ulceration 1 to 3 crypts; 2 = focal ulceration 3 to 5 crypts; 3 = focal ulceration more than 5 crypts; + 0.5 multifocal ulceration; + 1 multifocal ulceration more than 5 crypts. (**B**) Lamina propria haemorrhage and (**C**) lumen haemorrhage, scored as 0 ≤ 5 red blood cells (RBC); 1 = 6 to 10 RBC; 2 = 11 to 20 RBC; 4 = 21 to 50 RBC; 5 ≥ 51 RBC; +1 more than 3 foci of haemorrhage. (**D**) Neutrophilic infiltration of the lamina propria, determined as mean of ten 40x fields. (**E**) Mucosal thickness determined as mean of three measurements the crypts perpendicular to the mucosal surface. Each dot represents one pig. The lower, middle, and upper horizontal lines in the boxes correspond to the first quartile, median, and third quartile values. The lines extending above and below the boxes indicate the range of the upper and lower points within the 1.5 interquartile range. Results of Kruskal-Wallis test are indicated in the upper left corner of each panel. ns *P* > 0.05, * *P* ≤ 0.05, ** *P* ≤ 0.01, *** *P* ≤ 0.001, **** *P* ≤ 0.0001 (Wilcoxon test with Holm adjustment)

Further microscopic evaluation of the apex mucosa is summarized in Additional file 1. No lesions were observed in the apex from control pigs while intestinal mucosa damage increased in relation to disease severity. Thus, the apex from early infection group pigs showed epithelial erosion and mild inflammatory infiltration in the lamina propria while in the acute infection group, a complete loss of the epithelium, increase of mucosal thickness and greater neutrophils infiltration were observed (Fig. 2). The statistical analysis of this data revealed significant differences in ulceration among the three groups (*P* < 0.01). Significant differences between acutely infected pigs and either control or early infection groups were detected for the variables neutrophils infiltration (*P* < 0.001 and *P* < 0.05, respectively) and mucosal thickness (*P* < 0.001 and *P* < 0.001, respectively) (Fig. 3). No differences were observed in lumen haemorrhage while significant differences were detected in the lamina propria haemorrhage between acute infection and control groups (*P* < 0.01).

**Fig. 4:**
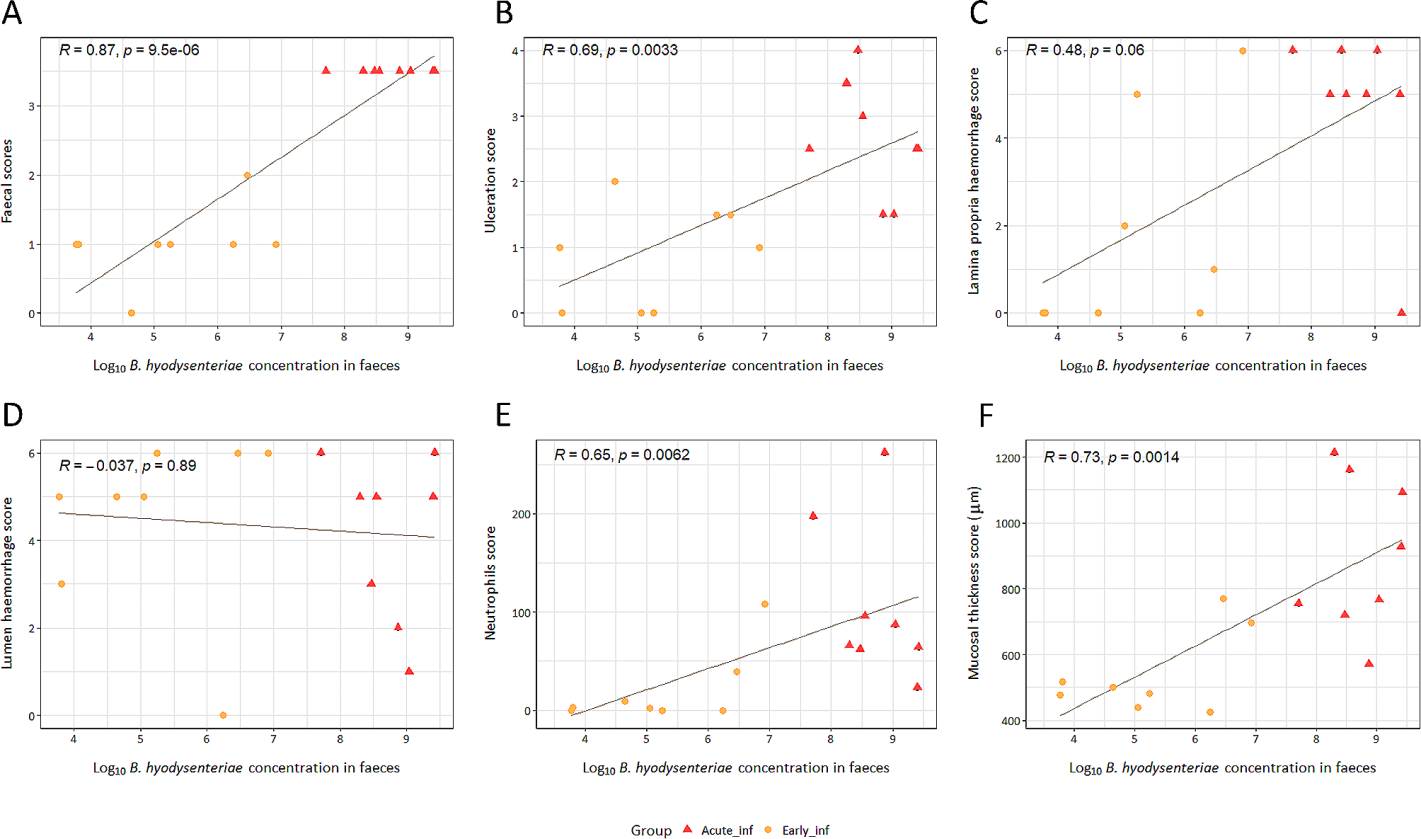
Correlation between concentration of *B. hyodysenteriae* in faeces and faecal score or microscopic lesions in infected pigs. Concentration of *B. hyodysenteriae* is estimated using q-PCR in last faeces before euthanasia. (**A**) Faecal score, last recorded before euthanasia (0 = normal, 1 = soft but formed, 2 = semisolid, 3 = liquid to watery, + 0.5 presence of mucus and / or blood) (Spearman’s rank order correlation). (**B**) Ulceration (0 = no ulceration; 1 = focal ulceration 1 to 3 crypts; 2 = focal ulceration 3 to 5 crypts; 3 = focal ulceration more than 5 crypts; + 0.5 multifocal ulceration; + 1 multifocal ulceration more than 5 crypts) (Pearson correlation coefficient). (**C**) Lamina propria haemorrhage and (**D**) lumen haemorrhage (0 ≤ 5 red blood cells (RBC); 1 = 6 to 10 RBC; 2 = 11 to 20 RBC; 4 = 21 to 50 RBC; 5 ≥ 51 RBC; +1 more than 3 foci of haemorrhage) (Spearman’s rank order correlation). (**E**) Neutrophilic infiltration of the lamina propria, determined as mean of ten 40x fields (Spearman’s rank order correlation). (**F**) Mucosal thickness determined as mean of three measurements the crypts perpendicular to the mucosal surface (Pearson correlation coefficient). Correlation coefficient with the *p*-value is indicated in the upper left corner and the line represent linear regression line of each panel

Similarly to faecal score, concentration of *B hyodysenteriae*, estimated by q-PCR in faeces, correlated positively with microscopic lesions in the apex (ulceration, neutrophils and mucosal thickness) (*R* = 0.69, *P* < 0.01; *R* = 0.65, *P* < 0.01 and *R* = 0.73, *P* < 0.01, respectively) (Fig. 4), thus, there was a link between disease intestinal severity and *B. hyodysenteriae* concentration in faeces.

### Potential biomarkers of swine dysentery

**Fig. 5:**
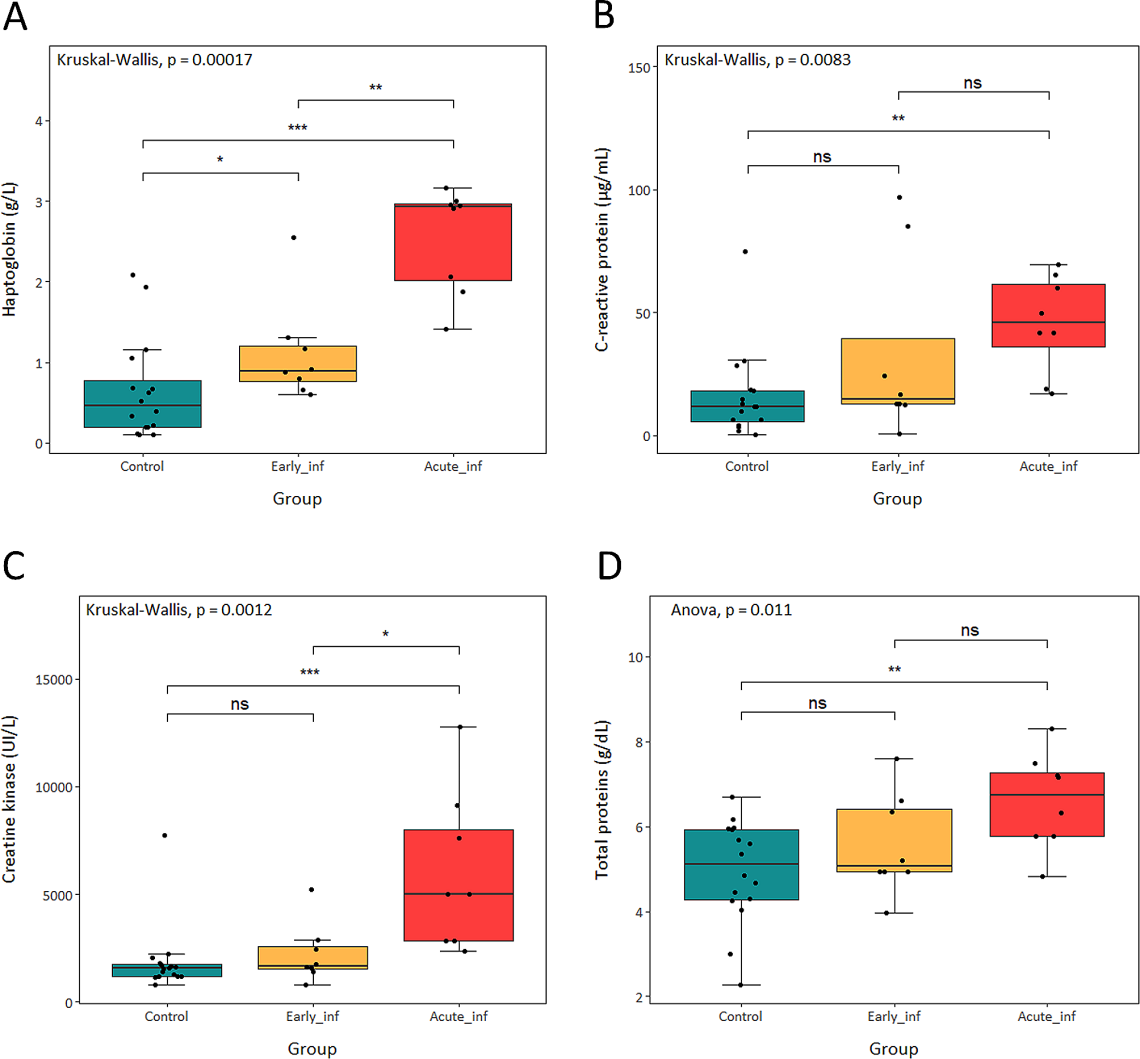
Boxplots of the measured serum biomarkers in controls and *B. hyodysenteriae* infected pigs. (**A**) Haptoglobin. (**B**) C- reactive protein. (**C**) Creatine kinase. (**D**) Total proteins. Each dot represents one pig. The lower, middle, and upper horizontal lines in the boxes correspond to the first quartile, median, and third quartile values. The lines extending above and below the boxes indicate the range of the upper and lower points within the 1.5 interquartile range. Results of Kruskal-Wallis (**A**, **B**, **C**) and ANOVA (**D**) test are indicated in the upper left corner of each panel. ns *P* > 0.05, * *P* ≤ 0.05, ** *P* ≤ 0.01, *** *P* ≤ 0.001, **** *P* ≤ 0.0001 (Wilcoxon (**A**, **B**, **C**) and Tukey (**D**) test with Holm adjustment)

The results for the array of 12 serum biomarkers are presented in Additional file 2 and Fig. 5. Among all potential biomarkers evaluated, only the biomarkers of inflammation, HAPTO and CRP, and the intestinal damage biomarker, CK, showed significant increase in pigs with mucohaemorrhagic diarrhoea compared to controls (*P* < 0.01, *P* < 0.01 and *P* < 0.001, respectively). In addition, HAPTO and CK values were also significantly higher in acute infected compared to early infected pigs (*P* < 0.01 and *P* < 0.05, respectively). The only factor with significant increased value in early infection, compared to non-infected animals, was HAPTO (*P* < 0.05). The PROT concentration also increased significantly (*P* < 0.01) in pigs with mucohaemorrhagic diarrhoea compared to non-infected pigs (Fig. 5). The other biomarkers tested, ALBU, TRIGL, ADA, BchE, AchE, PON 1, FRAP and Thiol, did not show any change associated to the infection.

The severity of the disease, defined by the microscopic lesions, faecal score, and pathogen shedding, showed a positive correlation with the part of the biomarkers tested (Additional file 3). The strongest correlations were observed among HAPTO, CRP and CK with faecal score (*R* = 0.719, *P* < 0.0001; *R* = 0.606, *P* < 0.001; *R* = 0.567, *P* < 0.001, respectively) and *B. hyodysenteriae* concentration in last q-PCR (*R* = 0.720, *P* < 0.0001; *R* = 0.576, *P* < 0.001; *R* = 0.571, *P* < 0.001, respectively). Regarding microscopic lesions, HAPTO and CRP showed strong correlation with lamina propria haemorrhage score (*R* = 0.589, *P* < 0.01 and *R* = 0.625, *P* < 0.001, respectively) and CK with ulceration score (*R* = 0.484, *P* < 0.01).

## Discussion

Despite the renowned impact of SD, a severe enteric disease characterized by mucohaemorrhagic diarrhoea with decades of scientific research beyond [1, 14], many aspects of its epidemiology still need further research.

With the aim of mimicking a natural infection as closely as possible, in our challenge, pigs were inoculated orally without intragastric gavage, and were fed a conventional diet during the challenge, an approach that differs from other experimental infections using *B. hyodysenteriae* [8–10, 13]. Compared to intragastric gavage, oral challenge does not require sedation, offering numerous advantages since sedated animal should remain lied in lateral recumbency and gastric motility may be reduced, hindering the infection. In addition, challenges usually provide diets with high protein concentration, (i.e., soy), which exceeds, by far, the protein percentage in regular diets for growers and finishers. This strategy facilitates the colonization of the pathogen by the microbiota disruption elicited [12]. In our challenge, pigs were inoculated orally for three consecutive days while receiving conventional diet and we reached a 100% of disease incidence (16/16), a better result than other studies using both, oral [11, 30, 31] or intragastric inoculation [9, 10, 13]. In contrast, the lapse between challenge and severe disease onset was longer than in studies using intragastric inoculation or high protein diets [8, 12]. It is worth to mention that factors such as strain, concentration used, animal genetics or age may affect the disease outcome as well. In agreement with the results of a recent study performed by Parra-Aguirre et al. [30], we can conclude that oral inoculation can be an effective method to experimentally reproduce SD without the use of sedatives and trained staff or diet induced dysbiosis.

The macroscopic pathological findings recorded were associated with the clinical signs observed, being more severe in pigs in acute disease, with mucohaemorrhagic diarrhoea, compared to pigs euthanized in early infection, with pathogen faecal shedding but with non-altered or soft faeces. In SD, the multiplication of *B. hyodysenteriae* in the large intestine causes progressive lesions, the mucosa becomes damaged and there is superficial haemorrhage and fibrinous exudation resulting, finally, in the characteristic mucohaemorrhagic diarrhoea [14]. We observed an exception for this axiom, as a pig (#PD5) euthanized in the early stage of the infection, with no records of diarrhoea, had a severely affected apex. Accordingly, Wilcock and colleagues [32] reported that the macroscopic or histologic severity of the lesion does not invariably increase with SD progression, having as much variation among sections from the same pig as among different pigs with different disease outcomes. It would be interesting to record the frequency of this phenomena under field conditions. Like previous studies [8, 14], macroscopic lesions in the intestinal tract were limited to the spiral colon, particularly in the apex.

It is well-known that at the onset of the disease, necrosis of intestinal epithelium develops rapidly without deeper penetration, while fibrosis and other changes appear when colitis persists over time [32]. For this reason, it is not surprising that ulceration was the only microscopic lesion we observed in the early infected pigs. In acute infected pigs in which the infection persists, pathogen multiplication leads to the disruption of the intestinal epithelium causing an exudative or inflammatory diarrhoea due to the outflow of plasma and blood into the intestinal lumen [33]. In addition, degeneration caused by persistent necrosis of epithelium prompts a hyperplasia of the crypts due to cell regeneration [32]. Altogether, these mechanisms explain the ulceration, lamina propria haemorrhage, and increased neutrophil counts and mucosal thickness observed in acute infected pigs.

Detection of *B. hyodysenteriae* in faecal samples relies on different techniques such as PCR and microbiological culture with studies describing no differences in sensitivity between these two methods [8]. We have monitored faeces daily by both, q-PCR and culture, and a higher sensitivity of q-PCR was evidenced. All challenged pigs were at some stage positive by q-PCR, but cultures were only positive when the concentration detected by q-PCR was higher than 10^5^ bacteria/g of faeces. Akase and colleagues [34] already highlighted the higher sensitivity of qPCR over traditional culture, fact that limits the usefulness of culture in early stages of the disease or in low concentrations of *B. hyodysenteriae* in faeces.

Faecal detection of *B. hyodysenteriae* usually occurs 0–3 days before diarrhoea onset [14, 35]. This period is consistent with our q-PCR results, but it can be prolonged in some individuals [8], as we observed in a single pig. These carrier animals are important from an epidemiological point of view, considering the increased opportunity for horizontal transmission prior to disease recognition. In addition, like previous studies, we also demonstrated association between *B. hyodysenteriae* concentration in faeces and severity of macro [32] or microscopic lesions [31]. Interestingly, in our study pigs shedding less than 10^5^ bacteria/g of faeces did not show lesions in the large intestine. Although further studies are required, we can propose this value as a cut-off to differentiate pigs with or without SD lesions.

In our study, APPs and other potential biomarkers were tested in serum samples from the three experimental groups made. No significant variations were observed among groups for markers of oxidative stress and TRIGL. Acute-phase proteins are produced as part of inflammatory processes and may be useful early-biomarkers for infectious diseases. In pig production, APPs have been used to distinguish healthy, subclinical and clinical diseased pigs [36]. APPs generally have a higher response in bacterial infections and in animals with clinical signs [15, 37], with several studies attempting to characterise APPs in SD [12, 14, 36, 38]. Among APPs, multiple studies have shown that HAPTO and CRP can be used to estimate pig health status [37, 39], together with serum amyloid A and the pig major acute phase protein [40, 41], although the last two were not measured in our study. HAPTO binds free haemoglobin e.g., released by haemolysis, which is toxic and proinflammatory, reducing oxidative damage [15], so it is used to monitor the occurrence and development of inflammatory conditions [42]. CRP plays important roles in protection against infection, clearance of damaged tissue and regulation of the inflammatory response, because it acts as an opsonin by binding to bacteria and free DNA [15, 42]. It has been reported that, under experimental infections, HAPTO increases rapidly and remains elevated for 10–15 days, whereas CRP shows a fast increase and declines after 4–5 days [41]. This fact may explain why in our study, the HAPTO concentration was significantly higher in acutely infected than in early infected pigs while the CRP concentration did not show this pattern. The significant increase observed in the acute infected group in PROT is not associated with an increase in serum ALBU levels, fact that preludes any association to pig dehydration and links these inflammation biomarkers to intestinal inflammation.

Determination of serum levels of intracellular enzymes is another appropriate method to evaluate intestinal damage [18]. As other studies have shown, CK is increased because of intestinal damage and necrosis [43, 44]. Therefore, is not surprising that CK levels were also significantly increased in acute infected pigs associated to SD severity.

Jacobson and colleagues [45] already hypothesized that the serum acute-phase response to SD was induced in response to intestinal lesions. This was also demonstrated by our results, in which the potential biomarkers HAPTO, CRP and CK significantly increased their concentration in acute infected pigs but with different degrees of correlation with the microscopic lesions found. There was not a strict relationship between the serum concentrations of these proteins and the severity of microscopic lesions, potentially explained by individual variations in cytokines expression, the main inducers of these proteins [45]. The fact that the main lesion observed in early infected pigs was ulceration may explain why only differences in HAPTO levels, the most sensitive indicator of acute inflammation [37, 46], were detected. According to our results, only HAPTO, CRP and CK could be linked to the presence of SD lesions in the large intestine, and just HAPTO could be detected in early infected pigs. The rapid decline of CRP precludes any conclusion about its usefulness as early biomarker. In summary, the results obtained revealed the association of certain serum APPs and SD severity, although, as mentioned in this study, APPs may be elicited by other pro-inflammatory processes and they do not replace microbiological detection methods.

## Conclusions

The present study adds information on *B. hyodysenteriae* transmission, diagnosis and clinical and lesion outcomes using a challenge model under controlled conditions. The study highlights the limitations in the diagnosis of *B. hyodysenteriae* using clinical signs, macro and microscopic lesions or microbiological culture, relying only in PCR results to detect shedders in early infection. In addition, the serum biomarkers tested did not provide any clear advantage over *B. hyodysenteriae* detection by qPCR. Finally, a correlation between faecal shedding and disease severity measured in intestinal lesions was demonstrated, a fact that allow us to propose *B. hyodysenteriae* quantification as useful tool to determine the severity of the disease on infected animals.

### Electronic supplementary material

Below is the link to the electronic supplementary material.

Supplementary Material 1

## Data Availability

No datasets were generated or analysed during the current study.

## Declarations

### Authors’ information

Lucía Pérez-Pérez (PRE2020-093762) is supported by the Spanish Ministry of Education.

## Abbreviations

AchE: Acetylcholinesterase
ADA: Adenosine deaminase
ALBU: Albumin
ANOVA: One-way analysis of variance
APPs: Acute phase proteins
ATCC: American Type Culture Collection
BchE: Butyrylcholinesterase
BHI: Brain heart infusion broth
CK: Creatine kinase
CRP: C-reactive protein
Ct: Cycle threshold
DPI: Days post-inoculation
FBS: Foetal bovine serum
FRAP: Ferric reducing ability of plasma, HAPTO: Haptoglobin
PBS: Phosphate-buffered saline
PON 1: Paraoxonase
PROT: Total protein
RBC: Red blood cells
SD: Swine dysentery
TRIGL: Triglycerides
TSA: Trypticase Soy Agar
