# Supplementary material for: New insights into swine dysentery: faecal shedding, macro and microscopic lesions and biomarkers in early and acute stages of Brachyspira hyodysenteriae infection
Source: Porcine Health Manag. 2024 Jun 29;10:24. doi: 10.1186/s40813-024-00375-9 (PMC11218200; doi:10.1186/s40813-024-00375-9)
Supplement: Supplementary file 1 — Supplementary Material 1 [file 40813_2024_375_MOESM1_ESM.docx]

**Additional file 1** Microscopic lesion scores of challenged pigs (early and acute infection) and non-infected controls in apex mucosa.

| Group | Pig | Ulceration^a^ | Lamina propria haemorrhage^b^ | Lumen haemorrhage^b^ | Neutrophils^c^ | Mucosal thickness (mm)^d^ |
| --- | --- | --- | --- | --- | --- | --- |
| Control | PC1 | 0 | 2 | 5+1 | 2 | 508.7233 |
|  | PC2 | 0 | 0 | 0 | 1 | 514.424 |
|  | PC3 | 0 | 0 | 5 | 7 | 637.3839 |
|  | PC4 | 0 | 0 | 5 | 1 | 408.2551 |
|  | PC5 | 0 | 2 | 1 | 6 | 563.0726 |
|  | PC6 | 0 | 2 | 0 | 4 | 486.3765 |
|  | PC7 | 3+1 | 0 | 5 | 0 | 441.298 |
|  | PC8 | 0 | 0 | 5 | 6 | 434.1589 |
|  | PC9 | 0 | 1 | 4+1 | 4 | 525.3807 |
|  | PC10 | 0 | 0 | 5 | 6 | 472.4646 |
|  | PC11 | 0 | 1 | 4 | 0 | 523.4339 |
|  | PC12 | 0 | 2 | 5+1 | 3 | 670.6443 |
|  | PC13 | 0 | 0 | 2+1 | 1 | 491.1118 |
|  | PC14 | 0 | 0 | 2+1 | 5 | 640.0888 |
|  | PC15 | 0 | 2 | 5 | 1 | 469.4629 |
|  | PC16 | 0 | 2 | 2+1 | 2 | 574.6986 |
| Early infection | PD2 | 2 | 0 | 5 | 9 | 501.1312 |
|  | PD4 | 0 | 2 | 5 | 2 | 439.8741 |
|  | PD5 | 1+0.5 | 0 | 0 | 0 | 425.4627 |
|  | PD9 | 1+0.5 | 1 | 5+1 | 39 | 769.9736 |
|  | PD10 | 1 | 5+1 | 5+1 | 108 | 697.9702 |
|  | PD11 | 1 | 0 | 4+1 | 0 | 478.5075 |
|  | PD12 | 0 | 4+1 | 5+1 | 0 | 481.7628 |
|  | PD13 | 0 | 0 | 2+1 | 3 | 516.5802 |
| Acute infection | PD1 | 1+0.5 | 5+1 | 1 | 87 | 767.8882 |
|  | PD3 | 2+0.5 | 0 | 5+1 | 64 | 1093.3541 |
|  | PD6 | 2+0.5 | 4+1 | 4+1 | 23 | 928.9116 |
|  | PD7 | 1+0.5 | 5 | 2 | 262 | 571.8704 |
|  | PD8 | 3+0.5 | 5 | 4+1 | 66 | 1213.0213 |
|  | PD14 | 3+1 | 5+1 | 2+1 | 62 | 720.2037 |
|  | PD15 | 2+1 | 4+1 | 5 | 96 | 1160.6871 |
|  | PD16 | 2+0.5 | 5+1 | 5+1 | 197 | 754.4703 |

^a^ Score: [0] no ulceration, [1] focal ulceration 1 to 3 crypts, [2] focal ulceration 3 to 5 crypts, [3] focal ulceration more than 5 crypts, [+0.5] multifocal ulceration, [+1] multifocal ulceration more than 5 crypts.

^b^ Score: [0] ≤ 5 red blood cells (RBC), [1] 6 to 10 RBC, [2] 11 to 20 RBC, [4] 21 to 50 RBC, [5] ≥ 51 RBC, [+1] more than 3 foci of haemorrhage.

^c^ Score mean of ten 40x fields.

^d^ Score mean of three measurements the perpendicular crypts to the mucosal surface.

**Additional file 2** Measurements of 12 serum biomarkers in controls and *B. hyodysenteriae* infected pigs (early and acute infection).

| Group | Pig | PROT (g/dL) | ALBU (g/dL) | TRIGL (mg/dL) | CK (UI/L) | CRP (µg/mL) | HAPTO (g/L) | ADA (U/L) | BChE (μmol/mL·min) | AChE (μmol/mL·min) | PON 1 (IU/mL) | FRAP (mmol/L) | THIOL (µmol/L) |
| --- | --- | --- | --- | --- | --- | --- | --- | --- | --- | --- | --- | --- | --- |
| Control | PC1 | 5.97 | 1.87 | 38.86 | 1141.3 | 74.7 | 1.94 | 17.62 | 0.3 | 0.5 | 33.5 | 0.2794 | 49.2 |
|  | PC2 | 4.25 | 1.96 | 27.24 | 7749 | 10.0 | 0.12 | 17.55 | 0.2 | 0.5 | 53.4 | 0.3167 | 74.5 |
|  | PC3 | 3.01 | 1.89 | 26.54 | 1654.9 | 3.3 | 0.39 | 25.90 | 0.3 | 0.6 | 61.8 | 0.2603 | 49.2 |
|  | PC4 | 5.93 | 2.04 | 56.25 | 1281.5 | 6.3 | 1.05 | 25.62 | 0.3 | 0.6 | 53.4 | 0.3089 | 33.0 |
|  | PC5 | 4.45 | 1.92 | 32.02 | 1632.7 | 2.0 | 0.19 | 21.32 | 0.1 | 0.5 | 41.5 | 0.4368 | 7.7 |
|  | PC6 | 5.60 | 1.90 | 34.77 | 790.9 | 28.4 | 2.08 | 21.49 | 0.2 | 0.5 | 47.4 | 0.3026 | 26.5 |
|  | PC7 | 2.27 | 1.25 | 59.56 | 1554.7 | 6.3 | 0.34 | 6.63 | 0.1 | 0.4 | 26.7 | 0.4240 | 40.8 |
|  | PC8 | 4.04 | 1.78 | 37.10 | 2054.9 | 0.3 | 0.10 | 46.45 | 0.4 | 0.6 | 45.4 | 0.7389 | 105.6 |
|  | PC9 | 4.86 | 1.95 | 33.98 | 1202.4 | 4.1 | 0.19 | 34.60 | 0.3 | 0.8 | 57.7 | 0.3647 | 41.3 |
|  | PC10 | 6.17 | 2.86 | 24.30 | 1401.4 | 11.7 | 0.21 | 45.61 | 0.3 | 0.5 | 62.2 | 0.4277 | 71.0 |
|  | PC11 | 5.35 | 2.04 | 107.81 | 1820.2 | 11.6 | 0.62 | 32.30 | 0.3 | 0.7 | 63.0 | 0.5040 | 42.6 |
|  | PC12 | 5.69 | 2.65 | 69.54 | 1208.1 | 18.1 | 0.10 | 5.40 | 0.4 | 0.9 | 71.8 | 0.4258 | 70.8 |
|  | PC13 | 4.67 | 2.08 | 51.25 | 2227 | 15.0 | 0.52 | 15.89 | 0.2 | 0.5 | 68.5 | 0.2972 | 49.1 |
|  | PC14 | 5.94 | 2.70 | 66.24 | 1596.2 | 18.6 | 0.68 | 36.22 | 0.4 | 0.6 | 76.4 | 0.4310 | 64.9 |
|  | PC15 | 4.30 | 2.40 | 87.83 | 1706.8 | 12.8 | 1.16 | 35.80 | 0.3 | 0.6 | 66.3 | 0.5758 | 69.8 |
|  | PC16 | 6.69 | 2.38 | 68.31 | 1201.8 | 30.6 | 0.67 | 37.48 | 0.7 | 1.0 | 68.5 | 0.4115 | 38.6 |
| Early infection | PD2 | 6.61 | 2.59 | 61.32 | 2463 | 0.8 | 0.87 | 41.54 | 0.4 | 0.8 | 68.8 | 0.6146 | 98.8 |
|  | PD4 | 3.97 | 1.40 | 34.68 | 1736.6 | 12.9 | 0.65 | 15.70 | 0.2 | 0.3 | 27.2 | 0.2194 | 22.0 |
|  | PD5 | 4.94 | 2.12 | 36.13 | 2894 | 12.4 | 0.61 | 20.40 | 0.2 | 0.5 | 58.0 | 0.3223 | 46.8 |
|  | PD9 | 5.20 | 2.33 | 80.26 | 804.4 | 16.9 | 0.80 | 28.39 | 0.4 | 0.9 | 30.6 | 0.5098 | 46.8 |
|  | PD10 | 4.94 | 1.83 | 61.22 | 1411.2 | 85.0 | 1.31 | 15.93 | 0.2 | 0.6 | 44.4 | 0.3584 | 31.6 |
|  | PD11 | 4.95 | 2.18 | 57.93 | 1621.4 | 12.9 | 1.17 | 18.92 | 0.3 | 0.5 | 57.9 | 0.4022 | 31.3 |
|  | PD12 | 7.60 | 3.00 | 71.36 | 1567.4 | 96.7 | 2.55 | 17.43 | 0.8 | 1.0 | 68.7 | 0.4103 | 65.6 |
|  | PD13 | 6.35 | 3.64 | 65.92 | 5222 | 24.4 | 0.92 | 43.99 | 0.4 | 0.8 | 66.4 | 0.4792 | 89.8 |
| Acute infection | PD1 | 5.76 | 2.35 | 30.54 | 5021 | 19.0 | 1.41 | 48.55 | 0.4 | 0.7 | 51.1 | 0.9954 | 207.1 |
|  | PD3 | 5.77 | 2.29 | 144.03 | 2369 | 69.5 | 2.95 | 28.42 | 0.2 | 0.5 | 50.0 | 0.4101 | 13.7 |
|  | PD6 | 6.31 | 2.28 | 43.85 | 2827 | 49.9 | 2.95 | 17.41 | 0.4 | 0.5 | 47.7 | 0.3199 | 37.4 |
|  | PD7 | 7.16 | 2.84 | 59.68 | 7605 | 41.8 | 3.01 | 19.44 | 0.1 | 0.3 | 44.8 | 0.3324 | 32.8 |
|  | PD8 | 4.82 | 1.90 | 35.06 | 4996 | 17.2 | 1.88 | 16.13 | 0.1 | 0.4 | 44.0 | 0.2480 | 26.4 |
|  | PD14 | 8.31 | 3.25 | 73.17 | 12766 | 41.6 | 3.17 | 26.84 | 0.3 | 0.4 | 66.1 | 0.3875 | 36.8 |
|  | PD15 | 7.49 | 2.85 | 65.94 | 9148 | 65.4 | 2.06 | 35.13 | 0.2 | 0.4 | 49.8 | 0.5746 | 87.8 |
|  | PD16 | 7.20 | 2.53 | 29.20 | 2846 | 60.1 | 2.91 | 32.27 | 0.5 | 0.7 | 49.5 | 0.3719 | 47.0 |

AchE: Acetylcholinesterase, ADA: Adenosine deaminase, ALBU: Albumin, BchE: Butyrylcholinesterase, CK: Creatine kinase; CRP: C-reactive protein, FRAP: Ferric reducing ability of plasma, HAPTO: Haptoglobin, PON 1: Paraoxonase; PROT: Total protein, TRIGL: Triglycerides

**Additional file 3** Correlation among serum biomarkers measurements with faecal score, *B.  hyodysenteriae* shedding and microscopic lesions in controls and *B. hyodysenteriae* infected pigs.

| Potential biomarkers | Microscopic lesions | | | | | Faecal score in last sample | *B. hyodysenteriae* concentration in last q-PCR |
| --- | --- | --- | --- | --- | --- | --- | --- |
|  | Ulceration score | Lamina propria haemorrhage score | Lumen haemorrhage score | Neutrophils score | Mucosal thickness score |  |  |
| HAPTO | **  R = 0.5327  P = 0.0017 | ***  R = 0.5858  P = 0.0004 | ns  R = 0.1549  P = 0.3972 | *  R = 0.4350  P = 0.0128 | *  R = 0.4113  P = 0.0193 | ****  R = 0.7191  P = < 0.0001 | ****  R = 0.7202  P = < 0.0001 |
| CRP | ns  R = 0.2902  P = 0.1071 | ****  R = 0.6251  P = 0.0001 | ns  R = 0.2722  P = 0.1318 | ns  R = 0.3469  P = 0.0517 | **  R = 0.5038  P = 0.0033 | ***  R = 0.6062  P = 0.0002 | ***  R = 0.5759  P = 0.0006 |
| CK | **  R = 0.4841  P = 0.0050 | ns  R = 0.1604  P = 0.3805 | *  R = -0.3555  P = 0.0458 | ns  R = 0.3262  P = 0.0685 | ns  R = 0.2698  P = 0.1352 | ***  R = 0.5670  P = 0.0007 | ***  R = 0.5705  P = 0.0007 |

CK: Creatine kinase; CRP: C-reactive protein, HAPTO: Haptoglobin.

ns P > 0.05, * P ≤ 0.05, ** P ≤ 0.01, *** P≤ 0.001, **** P ≤ 0.0001 (Spearman’s rank order correlation).
